# The Evolution of United States Governance Policies for Research Using Pathogens with Enhanced Pandemic Potential

**DOI:** 10.1089/apb.2024.0049

**Published:** 2025-06-05

**Authors:** Gerald L. Epstein

**Affiliations:** RAND Corporation, Arlington, Virginia, USA.

**Keywords:** biosafety, biosecurity, biorisk management, enhanced potential pandemic pathogens, gain-of-function, ePPP, oversight, pathogens with enhanced pandemic potential, PEPP, P3CO, research policy

## Abstract

**Background:**

Prompted by publications in 2012 involving the enhancement of a highly virulent but poorly transmissible human pathogen to make it more transmissible, the research community and the U.S. government have implemented policies to oversee research involving enhanced pathogens that pose the risk of causing a pandemic.

**Method:**

This article reviews the evolution of policies governing high consequence, government-funded research that has been called “gain-of-function-research-of-concern,” research with “enhanced potential pandemic pathogens” (ePPPs), and research with “pathogens with enhanced pandemic potential” (PEPPs). It analyzes features that these policies share and points out some of their shortcomings, challenges, and ambiguities.

**Results:**

These policies, culminating in the 2024 United States Government Policy for Oversight of Dual-Use Research of Concern and Pathogens with Enhanced Pandemic Potential, all define a set of consequential research activities that trigger the need for additional high-level review, and they all set out principles that must be satisfied before the research can be funded.

**Conclusion:**

The 2024 policy, like its predecessors, only applies to government-funded research. Extending it to cover privately funded research would require either new regulations under existing statutory authority or new legislation. Like its predecessors, the 2024 policy requires that the benefits of PEPP research justify its potential pandemic risk. Unlike its predecessors, however, the 2024 policy is missing an important principle that prevents construction of a pandemic pathogen that—were it not for its creation in the proposed research—would have little likelihood of ever causing an actual pandemic.

## Transmissible H5N1 Influenza Studies

In late 2011, the National Science Advisory Board for Biosecurity (NSABB)—a panel formed to advise the U.S. government about life science research done for legitimate purposes that could be misused for harm—deliberated over two draft scientific publications on high pathogenicity H5N1 avian influenza (HPAI H5N1). The disease, which was devastating bird populations, had a case fatality rate of over 50% in those few humans who had become infected by it,^1^ probably through direct contact with infected birds. Fortunately, the disease was not transmissible between humans. However, scientists and public health officials worried that the highly lethal disease might mutate into a form that was also highly transmissible between humans, posing an extremely serious pandemic threat.

The publications reviewed by the NSABB described experiments in which scientists succeeded in mutating the virus to make it transmissible by aerosol between ferrets, an animal commonly used to model influenza transmission among humans. The Board had no legal authority to control the publication of the articles or to mandate changes, but it was asked by U.S. government officials to review the articles and make recommendations about their disposition. The Board recommended that *Science* and *Nature—*the journals to which the drafts had been submitted—publish the articles, but only after redacting “certain details that could enable the direct misuse of the research by those with malevolent intent.”^2^ In a subsequent meeting in March 2012, the NSABB reversed its initial conclusion,^2^ voting unanimously to recommend full publication of an updated version of one of the articles^3^ and voting 12-6 to recommend full publication of a revised version of the other.^4^

The controversy, which attracted worldwide attention, revealed that the United States had no general policy in place to govern the conduct and dissemination of research to generate or enhance pathogens capable of causing pandemics.^a^ In addition to posing significant biosafety concerns, such research represented a particularly high-consequence example of a broader category called “dual-use research of concern,” defined by the NSABB 4 years earlier as:

Research that, based on current understanding, can be reasonably anticipated to provide knowledge, products, or technologies that could be directly misapplied by others to pose a threat to public health and safety, agricultural crops and other plants, animals, the environment, or materiel.^5^

Reviews of these publications opened up a fault line within the scientific community itself, with some scientists supporting the NSABB’s original recommendation to withhold sensitive details from publication^6^ and others urging that the research be published in its entirety.^7^

This article reviews the evolution of policy mechanisms that have been adopted by the virology community or implemented by various U.S. government agencies to oversee research of the type that was reviewed by the NSABB in 2011 and 2012 (Table 1). After having gone through several different names, the pathogens characterizing this category of research are now known as pathogens with enhanced pandemic potential (PEPPs).

**Table 1.** Governance policies discussed in this article

Date	Policy name	Issued/adopted by
January 2012	Voluntary moratorium on research involving highly pathogenic avian influenza H5N1 viruses leading to the generation of viruses that are more transmissible in mammals^8^	37 virologists
March 2012	U.S. Government Policy for Oversight of Life Sciences Dual-Use Research of Concern (2012 Federal DURC Policy)^19^	U.S. Government
February 2013	2013 HHS Framework to guide funding decisions for certain research with highly pathogenic H5N1 Avian Influenza (HHS H5N1 Framework)^9^	U.S. Department of HHS
August 2013	Extension of HPAI H5N1 Framework to include H7N9 Avian Influenza^21^	U.S. Department of HHA
September 2014	2014 United States Government Policy for Institutional Oversight of Life Sciences Dual-Use Research of Concern (2014 Institutional DURC Policy)^26^	White House Office of Science and Technology Policy
October 2014	2014 U.S. Government Gain-of-Function Deliberative Process and Research Funding Pause on Selected Gain-of-Function Research Involving Influenza, MERS, and SARS Viruses^12^	The White House
January 2017	2017 OSTP Recommended Policy Guidance for Departmental Development of Review Mechanisms for Potential Pandemic Pathogen Care and Oversight (P3CO) (2017 OSTP P3CO Guidance)^33^	White House Office of Science and Technology Policy
December 2017	2017 HHS Framework for Guiding Funding Decisions About Proposed Research Involving Enhanced Potential Pandemic Pathogens (2017 HHS P3CO Framework)^34^	U.S. Department of Health and Human Services
December 2022	Prepare for and Respond to Existing Viruses, Emerging New Threats, and Pandemics (PREVENT Pandemics) Act (Public Law 117-328. Division FF, Title II)^a^	Legislation passed by Congress and signed into law
May 2024	United States Government Policy for Oversight of Dual-Use Research of Concern and Pathogens with Enhanced Pandemic Potential (2024 OSTP DURC/PEPP Policy)^44^	Executive Office of the President

^a^ Public Law 117–328. Division FF, Title II.

HHS, Health and Human Services.

## 2012 Voluntary Moratorium

The primary authors of the two articles reviewed by the NSABB, along with 37 of their colleagues, defended the safety and the importance of their research. However, acknowledging the “intense public debate” that their studies had generated, they agreed in January 2012 to a 60-day pause in the type of research that the NSABB was evaluating.^8^ Specifically, this pause covered “any research involving highly pathogenic avian influenza H5N1 viruses leading to the generation of viruses that are more transmissible in mammals.” These researchers also stated that “no experiments with live H5N1 or H5 HA reassortant viruses already shown to be transmissible in ferrets will be conducted during this time.”^8^ The pause was intended to provide time “to find the best solutions for opportunities and challenges that stem from the work.”

The pause was extended for almost eight more months, at which point three of the principal avian influenza researchers called for the research to resume. They stated that the conditions for resuming research “have been met in some countries and are close to being met in others.”^17^ One year after the publication of the original moratorium announcement, the 39 original authors plus 1 additional signatory declared an end to the voluntary moratorium.^18^

## 2012 U.S. Government Policy for Oversight of Life Sciences Dual-Use Research of Concern (2012 Federal DURC Policy)

Shortly after the March 2012 NSABB meeting that recommended unredacted publication of the two articles described above, the United States Government released the U.S. Government Policy for Oversight of Life Sciences Dual-Use Research of Concern (DURC).^19^ This policy did not directly address the controversy over HPAI H5N1 research, but rather resurrected the NSABB’s 2007 recommendation to establish a review process for DURC more broadly.^5^ Under this policy, particular scrutiny would be given to proposals for federally funded research with any of a list of 15 high-consequence pathogens or toxins (which included HPAI H5N1) that was reasonably anticipated to manifest any of seven experimental effects (which included enhancing the harmful consequences, or increasing the stability, transmissibility, or dissemination ability, of an agent or toxin). Research satisfying both the agent and the experimental effect criteria would be reviewed to see if it met the definition given above of DURC. If it did, the policy called for the prospective funding agency to assess the proposed research’s risks and benefits and to review a mitigation plan to address those risks. If the risks could not be sufficiently addressed, one possible mitigation option would be to not fund the research, even if it was otherwise scientifically meritorious.

The 2012 DURC policy only applied to unclassified research. Given that a primary concern about DURC was that the information it generated could be misused for harm, any DURC research that was being conducted in a classified environment would already have controls on the resulting information.

This policy would have captured the HPAI H5N1 research that had come before the NSABB had it been in place when that research was first proposed, since HPAI H5N1 is one of that policy’s 15 high-consequence agents and increased aerosol transmissibility is one of its seven experimental effects. However, the policy’s scope was both broader and shallower than the issues raised by the NSABB’s 2011 and 2012 meetings. Broader, because this new oversight policy covered agents and experimental outcomes that extended considerably beyond enhancing the human-to-human transmissibility of HPAI H5N1. Shallower, because research that could conceivably induce the extraordinarily high consequences of a pandemic was held to no higher a standard than other types of DURC that risked less severe consequences.

## 2013 Department of Health and Human Services Framework to Guide Funding Decisions for Certain Research with Highly Pathogenic H5N1 Avian Influenza (HHS H5N1 Framework)

One of the factors influenza researchers pointed to in calling for an end to their self-imposed moratorium was a workshop held at the National Institutes of Health (NIH) in December 2012. At this workshop, NIH solicited feedback from members of the international influenza research community on a draft framework it had developed to govern decisions to fund certain research proposals with HPAI H5N1. NIH proceeded to issue the framework on February 21, 2013 (Department of Health and Human Services [HHS] H5N1 Framework).^9^

This framework formed the basis for all future U.S. government policies regarding research with potential pandemic viruses that had the effect of enhancing their pathogenicity and/or transmissibility. Structurally, they all consist of:

1. Specification of the type of research that requires higher level or departmental review, called the “trigger” in this article
2. Principles that all reviewed research must satisfy, and
3. Review above the level of the funding agency to determine whether proposals that satisfy the required criteria should indeed be funded, and under what conditions

The trigger language describing research requiring review under the 2013 HHS H5N1 Framework (see Box 1, p. 5 of reference 9) is that it is “reasonably anticipated to confer gain-of-function attributes that enable influenza viruses expressing the virulent form of the hemagglutinin (HA) gene from highly pathogenic H5N1 to be transmissible among mammals by respiratory droplets.”^9^ “Gain-of-function,” as used here, referred to studies of viruses that enhance transmission or virulence in mammals or alter the virus’s host range. In general, however, “gain-of-function” refers much more broadly to mutations in an organism that give it new or enhanced properties; the term is not restricted to enhancing virulence or transmissibility, or to conferring or enhancing a pathogen’s pandemic potential (see footnote 5, page 2 of the HHS H5N1 Framework). This ambiguity in the phrase would complicate U.S. policy and public debate for the next decade.

Box 2 (p. 5) of the 2013 HHS H5N1 Framework^9^ sets out the principles or criteria that must be satisfied by research to enhance influenza viruses in ways that are captured by that Framework's trigger language:

1. Such a virus could be produced through a natural evolutionary process;
2. The research addresses a scientific question with high significance to public health;
3. There are no feasible alternative methods to address the same scientific question in a manner that poses less risk than does the proposed approach;
4. Biosafety risks to laboratory workers and the public can be sufficiently mitigated and managed;
5. Biosecurity risks can be sufficiently mitigated and managed;
6. The research information is anticipated to be broadly shared in order to realize its potential benefits to global health;
7. The research will be supported through funding mechanisms that facilitate appropriate oversight of the conduct and communication of the research.

These criteria are mandatory; they are not merely recommendations to be considered. Of these, the first is the most important. It might be called the “No Blue Sky” principle, since in effect it states that pathogens should not be constructed if they have been dreamed up “out of the blue sky”^b^—in other words, if it would be extremely surprising for them to be the source of a future disease outbreak. Under such circumstances, research with such pathogens would pose considerable risk, given the possibility that they might escape confinement and initiate a pandemic, yet that research would have little benefit, since the disease that the research would be intended to counter would be very unlikely to arise otherwise. In fact, the only way that pathogen would ever be likely to pose a disease threat would be through a biosafety or biosecurity failure of the research program itself.

The other principles are to ensure that the research is scientifically meritorious; that it represents the lowest risk means of gaining information that is important to public health; that it can be performed, overseen, and communicated safely and responsibly; and that the benefits of having done the research will be made available to all. Interestingly, the principles do not contain an explicit statement that the benefits of the proposed research need to exceed the risks, although the narrative accompanying these principles does say that “evaluation of whether the risks outweigh the benefits” is to be part of the risk assessment process.

Research proposals meeting the trigger specification are to be reviewed at the departmental level, above the level of the agency that is considering funding it. This higher-level review is intended to provide some measure of independence, mitigating real or perceived conflicts of interest on the part of the funding agency. In addition to validating that all of the principles are satisfied, departmental review is to consider the risk assessments that have been prepared for the proposed research and the mitigation measures to address those risks; provide any additional or multidisciplinary expertise that may be needed, including in the areas of medical countermeasures, national security, law, public health preparedness, and response; and evaluate the proposal in the context of all other HHS research on HPAI H5N1 research. A negative departmental review decision prevents the proposal from being funded; a positive decision allows (but does not mandate) the funding agency to fund it.

## Extension of HPAI H5N1 Framework to Include H7N9 Avian Influenza

In early 2013, while the HPAI H5N1 framework was being prepared and rolled out, Chinese health authorities reported human fatalities from H7N9 avian influenza A.^20^ Fortunately, like HPAI H5N1, this strain of influenza was not very transmissible person-to-person. However, also like HPAI H5N1, there were concerns that a pandemic could result should the virus mutate to a more transmissible form. On April 19, the U.S. Secretary of Health and Human Services declared that “there is a significant potential for a public health emergency” involving H7N9 avian influenza A.^10^ In that context, HHS expanded its H5N1 HPAI framework to include research with H7N9 avian influenza A in August 2013.^21^

## 2014 Biosafety Lapses

In 2014, the United States’ premier biological laboratories—the National Institutes of Health and the Centers for Disease Control and Prevention—reported biosafety lapses regarding shipment of live, instead of inactivated, samples of *Bacillus anthracis*; the discovery of vials of the virus responsible for smallpox that were supposed to have been removed or destroyed 35 years previously; and the accidental contamination of low-pathogenicity cultures of avian influenza with the deadly H5N1 strain.^22^ In response, the U.S. government initiated a series of biosafety and biosecurity reviews. It also called for government life science labs to perform a “Safety Stand-Down” during which researchers and institutions were to review their safety protocols and procedures. U.S. government-funded laboratories were encouraged to do the same.^11^

The incidents did not involve the modification or construction of potential pandemic pathogens, the subject that had attracted so much attention 2 years previously. However, they called new attention to the risk of biosafety lapses, particularly on the part of major U.S. government laboratories whose biosafety and biosecurity protocols and procedures should have been the most thoroughly developed of any. They were also a pointed reminder of the potential consequences of biosafety lapses that might take place during research on pandemic pathogens.

A group of scientists calling itself the Cambridge Working Group issued a statement in the wake of the incidents urging that “creation of potential pandemic pathogens should be curtailed until there has been a quantitative, objective and credible assessment of the risks, potential benefits, and opportunities for risk mitigation, as well as comparison against safer experimental approaches.”^23^ A second group called “Scientists for Science,” which included the two principal investigators of the studies reviewed by the NSABB in 2011 and 2012, issued a statement countering that “biomedical research on potentially dangerous pathogens can be performed safely and is essential,” and that since the results of such research are often unanticipated, “risk-benefit analyses are difficult to assess accurately.”^24^

## 2014 United States Government Policy for Institutional Oversight of Life Sciences Dual-Use Research of Concern (2014 Institutional DURC Policy)

On September 24, the White House released the 2014 United States Government Policy for Institutional Oversight of Life Sciences Dual-Use Research of Concern, which covered institutions receiving Federal funds for life science research.^25,26^ Whereas the 2012 Federal DURC Policy addressed the funding of DURC research, this policy addressed how institutions needed to oversee it. This release was not a response to the 2014 biosafety lapses, but rather continued a policy process initiated in 2013 (prior to those lapses) when a draft version of the policy had been published for public comment.^27^ The definitions in the 2014 Institutional DURC policy mirror those in the 2012 Federal DURC policy, and like the 2012 policy, they would have captured the HPAI H5N1 research reviewed by the NSABB in 2011 and 2012 had the 2014 policy been in effect at the time.

## 2014 Funding Pause on Certain Gain-of-Function Research and Ensuing Deliberative Process

Unlike the 2014 Institutional DURC policy, which was not a response to the biosafety lapses earlier in the year, the U.S. government’s October 17, 2014 announcement of a funding pause on certain types of so-called “gain-of-function research” was a response to those lapses. The pause covered “gain-of-function research projects that may be reasonably anticipated to confer attributes to influenza, MERS, or SARS viruses such that the virus would have enhanced pathogenicity and/or transmissibility in mammals via the respiratory route,” and it was to remain in effect “until a robust and broad deliberative process is completed that results in the adoption of a new United States government gain-of-function research policy.”^12,29^

The pause explicitly applied to all U.S. government research, even though only HHS had funded the specific research projects that had come before the NSABB. This broad scope eliminated any perception that other government agencies might be able to fund research that HHS was precluded from funding. However, the pause contained a waiver—the heads of U.S. government funding agencies were permitted to proceed with research that would otherwise be blocked if they determined that the research was “urgently necessary to protect the public health or national security.” The qualifier “urgently” is important—even if the research were deemed “necessary,” it would not be eligible for an exception if it could wait until whatever approval process that would allow the pause to be lifted had been put into place.

The deliberative process called for by the funding pause included two international symposia by the National Academies of Sciences, Engineering, and Medicine (NASEM) (held in December 2014^28^ and March 2016^30^), a risk-benefit analysis conducted by Gryphon Scientific, a private contractor (delivered in April 2016),^13^ and a study by the NSABB with recommendations for the oversight of gain-of-function research (NSABB 2016, delivered in May 2016).^31^ The NSABB’s recommendations—together with rest of the record of the deliberative process, including the NASEM meetings, the risk/benefit analysis, and public comment—fed into an interagency process, overseen by the White House Office of Science and Technology Policy (OSTP), to develop U.S. policy to oversee this type of research.

## 2016 NSABB Recommendations for the Evaluation and Oversight of Proposed Gain-of-Function Research

The NSABB’s May 2016 recommendations proposed that additional review be conducted for research involving what NSABB termed “gain-of-function-research-of-concern” (GOFROC) (see page 2 of NSABB 2016).^31^ Such research would undergo a pre-funding review process that was modeled on the 2013 HHS H5N1 Framework.

Rather than depending on the use of specific pathogens, as did both the 2013 HHS Framework and the 2014 funding pause, the NSABB’s trigger language defining GOFROC was a functional one. It covered research that was “reasonably anticipated” to generate pathogens satisfying both of the following conditions:

- The pathogen generated is likely highly transmissible and likely capable of wide and uncontrollable spread in human populations (see page 41 of NSABB 2016).^31^
- The pathogen generated is likely highly virulent and likely to cause significant morbidity and/or mortality in humans (see page 42 of NSABB 2016).^31^

This functional definition was a significant advance in dual-use research oversight policy. For the first time, research to be covered by the policy was defined in a risk-based, rather than a list-based, way. Furthermore, the policy’s application depended on the properties of the pathogen that was to be *generated* in the course of the research, not those of whatever organism was used as the starting point.

In this formulation and in subsequent ones using similar language, some researchers objected to the “reasonably anticipated” language on the grounds that by definition, the properties of a pathogen produced in the course of a research project cannot be known in advance. (If they could be, the research would not be necessary.) On the other hand, such an anticipatory approach is a necessary aspect of any policy intended to identify and mitigate risks that may result from the conduct of an experiment that has not yet been done. It makes no sense to have to complete a research project in order to know if, or under what conditions, that project should have been done in the first place. For these purposes, the only alternative to “reasonably anticipating” the properties of a pathogen to be produced in the future is to “unreasonably anticipate” them.

The NSABB recommendation explicitly stated that “any study involving the generation of a pathogen exhibiting the two attributes above would be considered GOFROC.” However, as a way of baselining the types of studies that would tend not to need this type of additional review, the NSABB listed three categories of research that it “generally anticipated” would not constitute GOFROC:

- Studies to characterize the virulence and transmission properties of circulating pathogens
- Surveillance activities, including sampling and sequencing
- Activities associated with developing and producing vaccines, such as generation of high-growth strains (see page 42 of NSABB 2016)^31^

Like the 2013 HHS H5N1 Framework, the NSABB’s recommended approach listed several principles that any GOFROC research proposal would have to satisfy before being eligible for funding. Some, but not all, of the principles laid out by the NSABB are very similar to ones from the HHS H5N1 Framework. The full list of principles (see pages 43–44 of NSABB 2016)^31^ is:

1. The research proposal has been evaluated by a peer-review process and determined to be scientifically meritorious, with high impact on the research field(s) involved.
2. The pathogen that is anticipated to be generated must be judged, based on scientific evidence, to be able to arise by natural processes.
3. An assessment of the overall potential risks and benefits associated with the project determines that the potential risks as compared to the potential benefits to society are justified.
4. There are no feasible, equally efficacious alternative methods to address the same scientific question in a manner that poses less risk than does the proposed approach.
5. The investigator and institution proposing the research have the demonstrated capacity and commitment to conduct it safely and securely, and have the ability to respond rapidly and adequately to laboratory accidents and security breaches.
6. The results of the research are anticipated to be broadly shared in compliance with applicable laws and regulations in order to realize their potential benefits to global health.
7. The research will be supported through funding mechanisms that allow for appropriate management of risks and ongoing Federal and institutional oversight of all aspects of the research throughout the course of the project.
8. The proposed research is ethically justifiable.

The “No Blue Sky” principle has been carried over, with the additional requirement that there must be “scientific evidence” that natural evolutionary processes would be capable of generating the pathogen to be created in the experiment. Importantly, an explicit requirement that “the potential risks as compared to the potential benefits to society are justified” has been added as well. Even so, there is no requirement that those benefits be specific, realizable, and likely, which could open up the possibility that “potential” gains in knowledge might be viewed as justifying an arbitrary amount of risk. This danger is tempered by the “No Blue Sky” principle, which ensures that the pathogen to be created is one that might actually arise independent of the research, with the expectation that knowledge learned about it would have tangible value in preventing or countering its formation and spread.

All the other principles in the 2013 Framework had also been carried over, in some cases with slight modification. For example, the 2013 requirement that the research address “a scientific question with high significance to public health” was broadened to a requirement that it be peer-reviewed, scientifically meritorious, and high impact. The NSABB’s requirement that the research be “ethically justifiable” was also new.

## 2017 OSTP Recommended Policy Guidance for Departmental Development of Review Mechanisms for Potential Pandemic Pathogen Care and Oversight (P3CO)

With the May 2016 delivery of the NSABB’s recommendations, the OSTP initiated an interagency process to formulate a government policy that would lift the funding pause. This process led to publication on January 9, 2017, of the “Recommended Policy Guidance for Departmental Development of Review Mechanisms for Potential Pandemic Pathogen Care and Oversight (P3CO),” or the 2017 OSTP P3CO Guidance.^32,33^ Publication of this guidance did not itself lift the funding pause, but it specified the requirements for departmental review mechanism that, when implemented, would allow research funding that had been frozen by the 2014 pause to resume. The pause would not be lifted government-wide, but rather on a department-by-department basis as each department contemplating sponsoring such research formulated and implemented its own P3CO policy consistent with the OSTP guidance.

Like the 2014 funding pause, it was important for the OSTP P3CO Guidance to eliminate any perception that research in any part of the U.S. government was exempt from review. Therefore, the P3CO Guidance applied government-wide, and unlike the 2012 Federal DURC policy, it was not limited to unclassified research. This comprehensive, all-government approach should not be taken to imply that other agencies had been funding such research all along, that they anticipated doing so, or that any such research was being conducted in classified channels. Rather, it recognized that if future circumstances—perhaps ones that are not foreseeable today—called for agencies other than HHS to fund such research, those agencies should be subject to the same rules. The P3CO Framework was not intended to constitute either a blank check or a categorical ban.

Structurally, the OSTP P3CO Guidance followed the NSABB’s recommendations quite closely, and in fact, it stated that Federal departments and agencies should establish review mechanisms that were “generally aligned” with the NSABB’s approach. The trigger for the application of higher-level (departmental) review was drawn directly from the NSABB report, although with different terminology that avoided the ambiguous and overly broad phrase “gain-of-function.” OSTP relied on the NSABB, a Federal Advisory Committee of non-governmental experts with deep technical expertise in the subject domains that were relevant to dual-use research policy, to categorize research that merited additional review in a way that captured those activities posing the highest risk while at the same time did not cast such a wide net as to seriously impede research that did not require such close scrutiny.

Instead of referring to “gain-of-function research of concern,” the OSTP P3CO Guidance referred to the creation, transfer, or use of enhanced potential pandemic pathogens (ePPPs). Enhanced PPPs and PPPs were defined as follows (with paragraphs numbered as they appear in the Guidance):

2.2. A potential pandemic pathogen (PPP) is one that satisfies both of the following:
  2.2.1. It is likely highly transmissible and likely capable of wide and uncontrollable spread in human populations, and
  2.2.2. It is likely highly virulent and likely to cause significant morbidity and/or mortality in humans.
2.3. An enhanced PPP is a PPP resulting from the enhancement of a pathogen’s transmissibility and/or virulence. Wild-type pathogens that are circulating in or have been recovered from nature are not enhanced PPPs, regardless of their pandemic potential.^33^

Use of the term ePPP allowed the guidance to be written without use of the phrases “gain-of-function” or “gain-of-function-of concern.” However, it was subject to its own misinterpretation, in that an enhanced PPP could readily be taken to be the result of enhancing something that was already a PPP. To the contrary—an ePPP was defined as “a PPP resulting from the enhancement of a pathogen’s transmissibility and/or virulence” [emphasis added], with no requirement that the original pathogen already be a PPP. Neither was there any requirement that the starting point be a human pathogen. Any pathogen—including an existing PPP—that was enhanced to the point that it met the designated transmissibility and virulence criteria was an ePPP. It had to end up as a human pathogen after enhancement, but it didn’t need to start as one.

The OSTP P3CO Guidance went on to echo the NSABB’s listing of research that was “generally anticipated” not to require further review. However, it did so in a way that contradicted the NSABB’s explicit recommendation that “any study involving the generation of a pathogen exhibiting the two attributes above [i.e., transmissibility and virulence]” would require review. Instead, the Guidance said that:

2.5. To the extent that transmissibility and/or virulence of PPPs are modified in the following categories of studies, the resulting pathogens are not considered to be enhanced PPPs for the purposes of this recommended policy guidance:
  2.5.1. Surveillance activities, including sampling and sequencing; and
  2.5.2. Activities associated with developing and producing vaccines, such as generation of high growth strains.^33^

Stating these two categories of research in this way exempted them from review, as contrasted with the NSABB’s use of them to illustrate activities that would generally not require review, but that would still need to be reviewed if they sufficiently enhanced a pathogen’s transmissibility and virulence.

The policy principles to be applied to the research captured by the OSTP P3CO Guidance’s trigger were similar, but not identical, to those recommended by the NSABB. Many of the changes could be explained by the fact that the OSTP Guidance was to apply government-wide and was not restricted only to the HHS.

The principles in the OSTP P3CO Guidance, numbered as in the Guidance, are:

3.1. The proposal or plan for such a project has been evaluated by an independent expert review process (whether internal or external) and has been determined to be scientifically sound
3.2. The pathogen that is anticipated to be generated by the project must be reasonably judged to be a credible source of a potential future human pandemic
3.3. An assessment of the overall potential risks and benefits associated with the project determines that the potential risks as compared to the potential benefits to society are justified
3.4. There are no feasible, equally efficacious alternative methods to address the same question in a manner that poses less risk than does the proposed approach
3.5. The investigator and the institution where the project would be carried out have the demonstrated capacity and commitment to conduct it safely and securely, and have the ability to respond rapidly, mitigate potential risks and take corrective actions in response to laboratory accidents, lapses in protocol and procedures, and potential security breaches
3.6. The project’s results are anticipated to be responsibly communicated, in compliance with applicable laws, regulations, and policies, and any terms and conditions of funding, in order to realize their potential benefit
3.7. The project will be supported through funding mechanisms that allow for appropriate management of risks and ongoing Federal and institutional oversight of all aspects of the research throughout the course of the project; and
3.8. The project is ethically justifiable. Non-maleficence, beneficence, justice, respect for persons, scientific freedom, and responsible stewardship are among the ethical values that should be considered by a multidisciplinary review process making decisions about whether to fund research involving PPPs.^33^

Principle 3.1 requires independent expert review but does not require, as the equivalent NSABB recommendation did, that this review be “peer review,” a process that is not used in all government research programs. Principle 3.2 retains the “No Blue Sky” principle in that it recognizes that the only justification for doing ePPP research is to be better positioned to mitigate or prevent a future pandemic—and that such research would have no protective or prophylactic value if it addressed pathogens that could not credibly cause a future pandemic. However, the Principle also recognizes that future pandemics might result from pathogens were not the result of natural evolution, so limiting ePPP research to pathogens that could have evolved naturally would be too restrictive. Even so, by requiring that any enhanced PPP be a “credible source” of a potential future human pandemic, Principle 3.2 eliminates the ability to construct one based purely on speculation.

Principles 3.3, 3.4, 3.5, and 3.7 are identical, or nearly identical, to their NSABB-recommended counterparts. Principle 3.6 calls for research results to be “responsibly communicated,” rather than “broadly shared,” recognizing that circumstances could exist in which broad sharing of research results might aggravate the possibility of misuse. Principle 3.8 elaborates on the NSABB’s call for ePPP research to be “ethically justifiable.”

As with the 2013 HHS H5N1 Framework, proposed research that was captured by the trigger language and that satisfied all of the stated policy principles would go through a departmental review process outside of the agency proposing to fund the research. Any proposal that was disapproved in this departmental review could not be funded. Proposals that were approved would be eligible for funding.

Like all the previous government policies described here, the OSTP P3CO Guidance, and any subsequent departmental guidance, only cover federally funded research. Paragraph 8.2 of the OSTP P3CO Guidance laid down a marker for the government to consider future policy approaches through which oversight could be applied to “all relevant research activities,” and not just those that were federally funded. In practice, new legislation, or new regulations issued under existing legislation (if any were applicable), would be required to do so. No Executive Branch policy action can affect activities funded and conducted by non-governmental entities in the absence of statutory authority.

## 2017 HHS Framework for Guiding Funding Decisions About Proposed Research Involving ePPPs (2017 HHS P3CO Framework)

In December 2017, HHS released departmental policy, consistent with the 2017 OSTP P3CO Guidance, for reviewing enhanced potential pandemic pathogen research (2017 HHS P3CO Framework).^34^ In so doing, it brought the 2014 funding pause to an end with respect to its own activities. No other government departments or agencies have issued an equivalent policy—a fact that could be misinterpreted to mean that other government agencies are free to conduct ePPP research without constraint. In fact, the 2014 funding pause remained in effect for any government department or agency that did not have a departmental P3CO review process that was consistent with the 2017 OSTP P3CO Guidance.^c^

The 2017 HHS P3CO framework adopted the trigger language, policy principles, and departmental review provisions set out in the OSTP P3CO guidance. Interestingly, while the OSTP policy principles were preceded by language stating that agency review mechanisms “should establish” that relevant projects “satisfy” all the principles, the HHS framework describes the principles as “criteria” that departmental review will be “based on,” and that such review will “consider the eight criteria … and additional relevant factors and information.” Such wording appears to leave room for treating these principles as objectives that might be traded off among one another, and not as requirements. It is not known whether this difference in wording had any practical effect on HHS approval decisions.

After the 2017 HHS framework went into effect, four research proposals were evaluated under it: two had been frozen under the 2014 funding pause and were submitted for HHS P3CO review in 2018, after the pause had been lifted; one was submitted in 2019; and one in 2024. The first two projects were approved with “changes to increase the potential benefits while decreasing risks.” The third, which had contained both ePPP and non-ePPP elements, was funded only for the non-ePPP aspects, and the fourth, which consisted mainly of ePPP work, was not approved.^14^ The fact that only four projects have undergone P3CO review in 7 years has been quite surprising to those who participated in the formulation of the U.S. government P3CO policy.^d^

Moreover, the low number of reviewed research protocols is not consistent with survey data collected by Gillum et al., who among other things asked biosafety professionals from a wide swath of research institutions whether their institutions conducted research “that may generate enhanced Potential Pandemic Pathogens.”^35^ Of the 361 responses to this question, 63 said “yes” (see Table 2, p. 7 and Supplement A of Gillum et al.). It is not clear how many institutions this corresponds to, since multiple respondents may have come from the same institution, but these answers would likely indicate that many tens of institutions were conducting ePPP research at the time of the survey—far more than the less than one ePPP project per year that have actually been reviewed by HHS. Either the respondents were not familiar with the actual definition of an ePPP and were assuming the category to be far broader than it actually is, or the vast majority of ePPP research is not being captured by the ePPP policy. Neither explanation is very reassuring, and further clarification is prudent.

## Controversy Over NIH-Funded Coronavirus Research at the Wuhan Institute of Virology

The COVID-19 pandemic originated in Wuhan, China, in late 2019 when a bat coronavirus became highly transmissible among humans. In almost every previous case in which the origin of a human pandemic involving a zoonotic agent—one capable of infecting both animals and humans—has been identified, the source has been a “spillover event” in which the pathogen has crossed from its animal host into humans. Many virologists conclude that the highly infectious virus SARS-COV-2 that is responsible for COVID-19 started the same way, with a bat coronavirus that had infected an intermediate animal host jumping into humans at a live animal market in Wuhan.^15^ However, Wuhan is also the home of the Wuhan Institute of Virology (WIV), a world-famous coronavirus research institute that has studied—and engineered—some of the closest known analogs of SARS-COV-2. This juxtaposition immediately raised questions as to whether SARS-COV-2 could have originated in a laboratory, and not in a Wuhan market. Five years after the pandemic’s emergence, there is no definitive evidence pointing either way, although there are ardent proponents on both sides.

Those considering a laboratory origin for SARS-COV-2 identified NIH-funded research at the WIV, done as part of the project “Understanding the Risk of Bat Coronavirus Emergence,” as possibly being relevant. Among other things, the WIV work involved constructing chimeric coronaviruses that were based on a WIV “backbone” virus but had spike proteins—the part of the virus responsible for binding to host cells—taken in from other bat coronaviruses. These viruses were then tested in mice that had been engineered to express human versions of the receptor protein to which the viral spike protein would attach.^36^ Ability to infect these so-called “humanized mice” was taken as a proxy for ability to infect humans.

NIH has stated that this particular research project could not possibly have generated SARS-COV-2, given the genetic difference between that virus and the viruses studied in the NIH-funded project.^37^ However, considerable controversy surrounded the question of whether or not that research should have been subject to the 2014 funding pause, which was in effect at the time the grant was first issued, or the 2017 HHS P3CO Framework, which was in effect when the grant was renewed. NIH had determined that the original grant was not subject to the funding pause, but it placed a condition in the grant that if any of the chimeric viral strains produced in the experiment grew in culture to a concentration more than 10 times that of the backbone (unmodified) strain, the WIV work must stop and be reported back to NIH.^38^^,^^e^ NIH similarly concluded that the grant renewal was not subject to the 2017 HHS P3CO Framework, but it included the same condition on viral growth in the renewal grant.

Disputes over NIH’s handling of the work it funded at the WIV came to a head in a shouting match between Senator Rand Paul and Director of the National Institute of Allergy and Infectious Diseases Dr. Anthony Fauci at a July 20, 2021, Senate hearing. Senator Paul insisted that NIH had funded “gain-of-function” research at the Wuhan lab, whereas Dr. Fauci was equally insistent that it had not. Whatever other differences existed between the two men, this particular argument can be traced to the ambiguous definition of “gain-of-function.” Senator Paul used it to refer to research that increased the pathogenic properties of a virus, which the Wuhan research did. Dr. Fauci used it to refer to research requiring P3CO review, which NIH had determined the Wuhan research did not. Some may disagree with NIH’s decision here, but given the subjective judgments necessary to interpret the trigger language in the P3CO policy, NIH was within its rights to have made that call.

## 2022 NSABB Review of ePPP Policy

The attention given to NIH-funded research at the WIV, and the broader questions regarding possible lab origins of COVID-19, revived interest in U.S. policies governing enhanced potential pandemic pathogen research. The NSABB had been previously tasked (in January 2020) to make recommendations on balancing security with transparency in the government’s communications on ePPP research, but that assignment was suspended to allow NSABB members to devote their time to work more directly related to responding to the emerging COVID-19 pandemic.

Two years later, in February 2022, HHS reconvened the NSABB and broadened its charge, asking it to review the government’s policies regarding both ePPP research and DURC. After holding two public engagement meetings (April 2022 for ePPP^39^ and June 2022 for DURC^40^), NSABB issued its preliminary recommendations at a meeting on September 21, 2022,^41^ and it released its final report on March 6, 2023 (NSABB 2023).^42^

### Trigger Language and Principles

The NSABB’s review was conducted in the midst of the COVID-19 pandemic, caused by a highly transmissible but only modestly virulent virus that nevertheless had devastating worldwide effects. Accordingly, in defining “potential pandemic pathogen,” NSABB recommended broadening the 2017 P3CO policy’s trigger language, which had referred to pathogens that were both “likely highly transmissible” and “likely highly virulent,” so that it would capture pathogens that were both “likely moderately or highly transmissible” and “likely moderately or highly virulent” (emphasis added). Perhaps to partially compensate for this broadening, it also recommended adding the additional qualifier that a PPP need also be “likely to pose a severe threat to public health, the capacity of public health systems to function, or national security”(see page 3, NSABB 2023).^42^ That provision might have been inferred for a PPP as defined in the 2017 Guidance, but it had not previously been explicitly stated.

Furthermore, NSABB also found that the need to review proposals for creating or using ePPPs should not depend on the purpose behind any such manipulations. Therefore, it recommended removing the exemption for surveillance and vaccine development or production activities that had been included in the 2017 OSTP P3CO Guidance. Moreover, although it did not recommend replacement language, the NSABB warned that the term “enhanced PPP” lacked clarity. Although it was defined as the result of enhancement of *any* pathogen that resulted in a PPP, it could be taken to imply that the starting point already had to be a PPP.

The NSABB also recommended minor, non-substantive changes in the wording of two of the 2017 P3CO policies’ principles.

### Other Recommendations

Besides its recommendations for the trigger language and principles for P3CO review, the NSABB recommended that the DURC and P3CO policies be combined in an integrated framework, echoing a recommendation in the 2016 NSABB report that the Federal Government had not accepted at the time. It further recommended that the role, responsibilities, and expectations for researchers and institutions performing P3CO reviews be better articulated. The 2015 Institutional DURC Policy had provided some of this elaboration for the 2012 Federal DURC policy, but no institutional counterpart existed for the 2017 P3CO policies. Additional NSABB recommendations called for greater transparency in P3CO review and for consideration, in U.S. government policies on the defense of food and agriculture, of the need for analogous review of research with enhanced agricultural pathogens that might pose threats to human health, food, the economy, or national security.

NSABB recommended that any federally funded ePPP research conducted at foreign institutions be subject to review and oversight procedures equivalent to those applied domestically. The 2017 P3CO policies had made no distinction between domestic and foreign recipients, so this recommendation did not necessarily call for a change in policy, but particularly given the controversies over NIH-funded research in China, the NSABB apparently felt the need to make this point explicitly. Finally, the NSABB made recommendations regarding the Federal government’s DURC policies that are beyond the scope of this article.

## 2022 PREVENT Pandemics Act Language on Enhanced Pathogens of Pandemic Potential

The first statutory reference to ePPPs occurs in Public Law 117–328 (Consolidated Appropriations Act, 2023, 12/29/2022) in a subsection titled the “Prepare for and Respond to Existing Viruses, Emerging New Threats, and Pandemics (PREVENT Pandemics) Act.”^f^ Section 2315 of that legislation (incorporated into the U.S. code as section 6627 of title 42) calls for the Director of OSTP to review and “establish or update a Federal policy for the consistent review and oversight” of proposals for federally funded research that may be “reasonably anticipated to involve the creation, transfer, or use of enhanced pathogens of pandemic potential.”^43^ It does not define “enhanced pathogens of pandemic potential,” but rather specifies that the policy clearly identify the scope of research proposals that would be subject to the policy. Without specifically referencing the principles listed in the existing P3CO policies, the legislation addressed many of them in calling for an oversight policy that:

• “Appropriately considers the risks associated with, and potential benefits of, such research” (related to principle 3.3 of the 2017 OSTP P3CO Guidance);
• “Accounts for safety, security, and ethical considerations” (related to principles 3.5 and 3.8);
• “Includes measures to enhance transparency and public availability of information related to such research activities in a manner that does not compromise national security, the safety and security of such research activities, or any identifiable, sensitive information of relevant individuals” (related to principle 3.6);
• Calls for proposals “that have been determined to have scientific and technical merit” to be identified and referred to review (related to principle 3.1), and
• Ensures that research subject to review is regularly monitored to ensure compliance with the policy, and that research not initially subject to review but that produces unanticipated results that would bring it within the policy’s scope are identified.

Although the statute could have conferred authority for the U.S. government to regulate all research with enhanced pathogens of pandemic potential, regardless of funding source, it—like the existing U.S. government P3CO policies—was restricted to federally funded research. However, within that domain, it authorized the OSTP Director to issue a government-wide policy that is binding on internal and external recipients of Federal funding. OSTP had not previously had that authority, explaining why the 2017 OSTP P3CO Guidance “recommended” policy guidance that departments and agencies “should develop,” rather than issuing those policies itself or mandating that agencies do so. OSTP’s actions in 2017 did not lift the 2014 funding pause so much as provide a way to determine when the conditions for lifting the pause that were established when the pause first went into effect—that a “robust and broad deliberative process is completed that results in the adoption of a new USG gain-of-function research policy”^12^—were met.

## 2024 OSTP DURC/PEPP Policy

On May 6, 2024—14 months after the NSABB released its recommendations—OSTP exercised the authority conferred to it in 42 USC §6627 (the PREVENT Pandemics Act) and released the “United States Government Policy for Oversight of Dual-Use Research of Concern and Pathogens with Enhanced Pandemic Potential” (2024 OSTP DURC/PEPP Policy).^44^ The policy is to come into force one year later, on May 6, 2025. This document integrated and superseded the 2012 Federal DURC policy, the 2014 Institutional DURC policy, and the 2017 P3CO policies, and it was accompanied by implementation guidance (OSTP Implementation Guidance).^45^ In addition to explicitly citing 42 USC §6627 as its underlying authority, the policy states that “affected departments and agencies concur with this Policy, and an appropriate official from each affected department or agency has committed the department or agency to fulfilling the responsibilities herein described to the extent consistent with applicable law.” In other words, even if OSTP did not have legal authority to compel agencies to abide by the policy, all of them have agreed to do so anyway. (This provision may be more pertinent to the DURC parts of the policy, which were not explicitly authorized by the PREVENT Pandemics Act.)

The new integrated policy addresses two types of research, “Category 1” and “Category 2.” Category 1 includes, but broadens, research that had been under the purview of the 2012 and 2014 DURC policies; it will not otherwise be addressed here. Category 2 includes, but broadens, research that had been governed by the P3CO policies.

### Trigger Language

The trigger language defining Category 2 research is quite complicated, but in at least one way it is considerably clearer. It eliminates the ambiguity in the phrase “enhanced potential pandemic pathogen” by reordering the words to form “pathogen of enhanced pandemic potential (PEPP).” (Moreover, the ambiguous, and for policy purposes unhelpful, phrase “gain-of-function” nowhere appears. This absence continues the precedent set by the 2017 OSTP and HHS guidance documents, which did not use it either except when quoting earlier documents.) A PEPP is defined as “a type of pathogen with pandemic potential (PPP) resulting from experiments that enhance a pathogen’s transmissibility or virulence, or disrupt the effectiveness of pre-existing immunity, regardless of its progenitor agent” (emphasis added).” The language on disrupting pre-existing immunity is new but is not a significant change, since disrupting existing immunity would have the effect of increasing virulence and/or increasing transmissibility. Moreover, in other new language that does not really represent a policy change, a footnote goes on to state that enhancing a pathogen’s transmissibility would be taken as including enhancing its environmental stability or broadening the types of tissue or species it could infect, if those changes increased the ability to infect and transmit between humans.

OSTP’s definition of PEPP goes on to include an additional criterion along the lines of the NSABB recommendation that the pathogens subject to enhanced scrutiny be ones capable of posing a “severe threat to public health, the capacity of health systems to function, or national security.”^43^ However, OSTP used “significant threat” rather than “severe threat.” Neither is quantified, but the bar set by the OSTP language would be lower—and the corresponding set of PEPPs larger.

OSTP’s definition of a PPP—which underlies the definition of a PEPP—is “a pathogen that is likely capable of wide and uncontrollable spread in a human population and would likely cause moderate to severe disease and/or mortality in humans.” The transmissibility part of this definition retains the wording about “wide and uncontrollable spread” from both the 2017 OSTP language and the 2023 NSABB recommendation. However, by dropping “likely highly transmissible” from the 2017 definition, it renders the NSABB’s recommendation to broaden “highly” to “moderately or highly” here moot. OSTP did accept that recommendation in changing “likely highly virulent” (2017 language) to “would likely cause *moderate to* [emphasis added] severe disease.” In both cases, only human disease was included.

In the 2017 OSTP P3CO Guidance, additional review was required for any research that would create, use, or transfer ePPPs. The 2024 policy defines PPP and PEPP in ways that are largely similar to the use of PPP and ePPP in the 2017 guidance, but it adds another layer of complexity in specifying the types of research that require further review. Perhaps to parallel the definition of Category 1 research, which lays out a three-part test involving the pathogens to be used, the experimental outcomes to be produced, and a determination that the research constitutes “Concern DURC,” the definition of Category 2 research is based on three criteria:

• It involves a PPP or “any pathogen that will be modified in such a way that is reasonably anticipated to result in a PPP”;
• It is “reasonably anticipated” to produce experimental outcomes involving enhanced transmissibility, enhanced virulence, enhanced immune evasion, or the generation or use of an extinct or eradicated PPP or a previously identified PEPP; and
• It is “reasonably anticipated to result in the development, use, or transfer of a PEPP or an eradicated or extinct PPP that may pose a significant threat to public health, the capacity of health systems to function, or national security.”

The third criterion does not add much. Since the definition of PEPP already requires that the agent pose a “significant threat” to those listed qualities, that criterion’s only effect is to exclude from Category 2 any research involving an eradicated or extinct PPP that would be judged NOT to pose that sort of “significant threat.” But since there would be little population immunity to an eradicated or extinct PPP, it seems that any research involving one would indeed pose such a threat.

Importantly, the 2024 trigger language adopts the NSABB’s recommendation to eliminate the 2017 P3CO policies’ exemptions for surveillance and vaccine-related research. Those objectives are worthy ones, but they are not infinitely so, and proposals to construct or use PEPPs in the course of such research should demonstrate—not blindly assume—that the associated risks are justified. These subject areas are described as “typically not within scope of Category 2 research,” but the policy is clear that that is because they typically do not involve enhancing pathogens in ways that would create PEPPs. Directing researchers to “exhibit vigilance and evaluate research in case unexpected results warrant Category 2 review” makes clear that these research areas are indeed covered by the guidance if they produce PEPPs.

Overall, Category 2 likely includes a broader set of research than what would have required additional review under the 2017 OSTP P3CO Guidance, but it does not appear to have swept in significant amounts of research that do not merit added scrutiny. However, the actual impact of the expanded definition will not be known until the policy comes into force in 2025.

### Policy Principles

The biggest surprise in the PEPP treatment within the 2024 OSTP DURC/PEPP policy is the deletion of the “No Blue Sky” principle from the list of principles that PEPP research must satisfy. The remaining principles are very similar to their 2017 counterparts, but in presentation they appear to have been devalued. Whereas they had been highlighted in a major “Policy Principles” section in the 2017 OSTP P3CO Guidance and called out in a stand-alone box in the 2017 HHS Framework, the 2024 policy presents them as unlabeled sub-sub-bullets of part E of section 5.3, “Responsibilities of Federal Funding Agencies.” At least there, they are introduced with language that eliminates any impression the 2017 HHS Framework might have conveyed that they were not mandatory—the 2024 policy prefaces them by saying that “the proposed [Category 2] research will satisfy [emphasis added] the following criteria” (see pages 22–23 of the 2024 OSTP DURC/PEPP Policy):^44^

• “It has been evaluated by an independent scientific merit review process (whether internal or external) through already established Federal funding agency review processes and has been determined to be scientifically sound and of merit” (similar to 2017 OSTP P3CP Guidance principle 3.1)
• “It has undergone an assessment of the overall potential risks and benefits associated with the research, which determines that the potential benefits to society justify the potential risks” (similar to 2017 OSTP P3CO Guidance principle 3.3 and generally not accepting the alternate language recommended for this principle in the 2023 NSABB report)
• “There are no feasible, equally efficacious alternative methods to address the same research question in a manner that poses less risk than does the proposed approach” (nearly identical to 2017 OSTP P3CO Guidance principle 3.4 and not accepting the 2023 NSABB recommended language for this principle)
• “The PI and the research institution where the research would be carried out have the demonstrated capability and commitment to conduct it safely and securely, and have the ability to respond rapidly, mitigate potential risks, and take corrective actions in response to laboratory accidents, lapses in protocol and procedures, and potential security breaches” (nearly identical to 2017 OSTP P3CO Guidance principle 3.5)
• “Its results are anticipated to be responsibly communicated, in compliance with applicable laws, regulations, and policies, and any terms and conditions of funding, in order to realize their potential benefit” (nearly identical to 2017 OSTP P3CO Guidance principle 3.6)
• “It will be supported through mechanisms that allow for appropriate management of risks and ongoing U.S. government and/or institutional oversight of all aspects of the research throughout the course of the project” (nearly identical to 2017 OSTP P3CO Guidance principle 3.7); and
• “It is ethically justifiable. Non-maleficence, beneficence, justice, respect for persons, scientific freedom, and responsible stewardship are among the ethical values that should be considered by the department-level multidisciplinary review entity” (nearly identical to 2017 OSTP P3CO Guidance principle 3.8)

The missing “No Blue Sky” principle, principle 3.2 of the 2017 OSTP P3CO Guidance, had been the most important one, as described earlier, and its absence is difficult to explain. It is possible that this gap will have no direct consequences, since the requirement for benefits to exceed risks remains. Research involving a PEPP that is not a credible source of a future pandemic poses considerable risk but has little expectation of conferring any benefit, since whatever is learned would—at best—only indirectly apply to a pandemic that might actually happen. However, lack of the “No Blue Sky” principle permits researchers to argue that the fundamental knowledge gained in such an experiment might someday prove so beneficial in countering a future pandemic that collecting it warrants the risk of creating a present one. Such a justification obviates the entire rationale for review, since it assumes that the unquantifiable value of fundamental research justifies any amount of societal or planetary risk. Moreover, the optics of having removed this principle, when the others are retained near-verbatim, might lead observers to wonder whether this criterion was removed because it got in the way of experiments that some agency wanted to do. Such an interpretation would be unfortunate.

Stating that the quest for fundamental knowledge should not be sufficient justification for assuming the risks of PEPP research is not equivalent to saying that fundamental research should in general be held to a strict risk/benefit test. Given that the long-term benefits of fundamental research are often unanticipated at the time the research is performed and might not be realized for years after the research has been completed, it is almost never reasonable to impose a strict risk/benefit test on it. Although the benefits are unquantifiable, they might be huge, with only the cost and effort devoted to the research being at risk. However, when the risks of the research could assume societal or planetary proportions, the need for specific, articulable benefits to exceed potential risks becomes paramount. That was what the “No Blue Sky” principle had recognized.

### Other Policy Aspects

Except for the deletion of the “No Blue Sky” principle, the 2024 OSTP DURC/PEPP policy improves upon its predecessors in a number of ways. As a single integrated, government-wide policy, it harmonizes three formerly distinct policies and makes clear that the entire U.S. government is bound by it. Whereas the 2017 OSTP P3CO Guidance encouraged agencies to provide visibility to experts from other agencies in their departmental reviews, and the 2017 HHS Framework allowed HHS to include non-voting participants from other agencies in them, the 2024 policy states that the funding agency, “will, consistent with applicable law, provide” relevant law enforcement and intelligence agencies the opportunity to share information that might bear on risk-benefit assessments (see Section 5.3E, page 22 of the 2024 OSTP DURC/PEPP Policy).^44^

The policy includes an emergency waiver provision that is an improvement over the exemption provision in the 2014 funding pause, which was unrestricted so long as the need was judged “urgently necessary,” as well as over the 2017 P3CO policies, which did not provide for any waivers at all (although any emergency sufficiently compelling to warrant urgent ePPP research should also have been able to command sufficient senior management attention for the necessary review to be conducted on an urgent basis as well). The 2024 policy provides for conditional waivers, allowing the head of a department or agency to temporarily exempt research from the policy’s oversight mechanisms if (1) the research is urgently required to respond to a declared Public Health or other emergency, (2) the suspension of oversight is necessary for an effective response to the emergency, and (3) benefits of the waiver exceed the risks. Any such waiver would expire in 180 days but may be renewed. The policy would have been even stronger if the waiver was one-time only, since 180 days should provide ample time to complete the necessary review for any urgently necessary research project.

Acknowledging concerns over the meaning of “reasonably anticipated,” the policy provides a definition:

‘Reasonably anticipated’ describes an assessment of an outcome such that, generally, individuals with scientific expertise relevant to the research in question would expect this outcome to occur with a non-trivial likelihood. It does not require high confidence that the outcome will definitely occur but excludes experiments in which experts would anticipate the outcome to be technically possible, but highly unlikely (see Section 3.M, page 9 of the 2024 OSTP DURC/PEPP Policy).^44^

Applying this definition still requires making informed judgments about the outcome of future research, but it provides additional guidance regarding who would be making such a judgment and what such a judgment would mean.

### Policy Gaps

The intent of the 2024 OSTP DURC/PEPP policy, like its preceding P3CO policies, is to exercise governance over the enhancement of pathogenic viruses that have, or are given, pandemic potential, without constraining research with unmodified viruses that have been recovered from nature. However, there are two types of enhancement that would not be covered, and consequently potential pandemic pathogens could be produced through either mechanism without undergoing the reviews that are called for in the policy. Fortunately, these gaps can be closed with very minor wording changes.

First would be the creation of a PPP by enhancing a non-pathogenic organism, which would not be covered because the trigger language of this policy refers to the modification of “any pathogen.” Changing “any pathogen” to “any microorganism” here would close this loophole.

Second would be the creation of a novel potential pandemic pathogen that was not based on an existing pathogen (or microorganism, if the change above is made). The policy captures the creation of an “eradicated or extinct” PPP, but not one that is created ab initio*,* or that at least cannot be characterized as a modification of any given organism. Biological design tools utilizing artificial intelligence might eventually make it possible to design and build such novel pathogens.^46^ Another possible mechanism for creating a novel pathogen was highlighted by a group of 38 scientists that recently called attention to the risks of creating so-called “mirror organisms.”^16^ Many of the organic molecules upon which all life on Earth is based, such as nucleic acids and proteins, can come in two different forms, which—like left and right hands—are mirror images of each other but are not identical. However, life on Earth uses only one of these two possible forms. The scientists warned that should “mirror organisms” be constructed whose molecules were solely of the opposite mirror form, they could likely evade human, animal, and plant immune systems, causing pervasive lethal infections. These would certainly qualify as PPPs.

Neither of these mechanisms is likely to be possible in the near term, but at least for logical completeness, the OSTP 2024 DURC/PEPP policy should be modified to cover them—and changing “eradicated or extinct” to “novel, eradicated, or extinct” would do so.

## Next Steps

Any policy issued by the U.S. government’s Executive Branch will necessarily be incomplete in at least two important areas: research funded by non-governmental sources and research funded abroad. Only statutes passed by Congress can constrain what entities outside the U.S. government do with their own funds. And while the U.S. government can set an example for other governments to follow suit, the U.S. government has no authority over the activities of other countries. Yet PEPP research conducted by the private sector or overseas can have just as much impact on the United States—and the rest of the world—as PEPP research funded and/or conducted by the U.S. government.

### International Engagement

The 2017 OSTP P3CO Guidance recognized its own limitations in both of these areas. Section 7.3 on “International Engagement” states that the U.S. government “should engage with other countries about policies concerning creation, transfer, and use of enhanced PPPs, encouraging the development of harmonized policy guidance,” although it does not call for specific steps or establish specific milestones for doing so. Working with international partners, whether bilaterally, multilaterally, or within existing international institutions, could be useful in encouraging the development of analogous policies around the world. Non-governmental institutions such as national academies of sciences, professional associations, and civil society organizations can play a major role here as well. Indeed, the International Biosecurity and Biosafety Initiative for Science, or IBBIS, was formed to foster the harmonized development of biosafety and biosecurity governance mechanisms around the world.^g^

### Legislation

The 2017 OSTP P3CO Guidance also implicitly acknowledged its own inability to reach beyond government-funded research by calling for the future consideration of “other mechanisms that would enable oversight of all relevant research activities, regardless of the funding source.”^33^ Those “other mechanisms” would have to involve legislation, a route that the U.S. government has traditionally been reluctant to apply to scientific research.^47^

Although legislation is necessary to capture non-government-funded PEPP research, it does not necessarily need to be new legislation; the existing select agent statute might provide the means to do so (United States Code, Title 42, section 232a). This law authorizes the Secretary of Agriculture and the Secretary of Health and Human Services to impose regulatory controls over biological agents and toxins that have “the potential to pose a severe threat to public health and safety.” Existing select agent pathogens are either specified in regulation by name (e.g., “*B. anthracis*”) or by a specific description (e.g., “SARS-CoV/SARS-CoV-2 chimeric viruses resulting from any deliberate manipulation of SARS-CoV-2”).^48^ It is possible that PEPPs could be added to the list of select agent pathogens, defining them functionally in terms of their virulence, transmissibility, and enhancement, along the lines given in the 2024 OSTP DURC/PEPP policy. Such an action would bring them under regulatory control regardless of how their use was funded. However, this definition is, to some degree, inherently subjective, which could violate the Constitution’s due process requirements if courts were to rule that it did not provide sufficient clarity for researchers to understand what activities were permitted and what were prohibited.^47^

Designating PEPPs as select agents would allow their creation or use to be designated as “restricted experiments,” which are banned “unless approved by and conducted in accordance with the conditions prescribed” by the Secretary.^49^ Basing those conditions on the relevant portions of the 2024 OSTP DURC/PEPP policy would in effect broaden that policy to bring all such research into a legally binding regime.

If bringing PEPP research into the select agent program was deemed infeasible or undesirable, the only way to subject them to regulatory control would be through new legislation, which would describe the activities to be regulated and empower a Federal agency to develop the necessary regulations. Legislation can be amended only by subsequent legislation, which future Congresses cannot be presumed to be willing to pass, whereas regulations can be updated at the discretion of the regulatory agency. Therefore, any provisions of such a legal control regime that might change as science advances, or that might need to be adjusted as assessed risks and benefits of such research were to change, would more appropriately be specified in regulation rather than law.

## Conclusion

A pair of scientific publications in 2012 that involved the enhancement of a highly virulent but poorly transmissible human pathogen to make it transmissible through aerosol means attracted a considerable amount of controversy, both within the scientific community and among the broader public. In response, the U.S. government has developed several iterations of policy overseeing research that has been variously called “GOFROC,” research with “ePPPs,” or research with “PEPPs.” This evolution of policy has culminated in the 2024 issuance of the United States Government Policy for Oversight of Dual-Use Research of Concern and Pathogens with Enhanced Pandemic Potential, which will come into effect in May 2025.

The 2024 policy integrates the framework for PEPP research with policies that address broader but less consequential categories of pathogen research, establishing a single comprehensive, government-wide approach. However, it omits one of the most important principles from its predecessors—one that had precluded research to construct potential pandemic pathogens unrelated to ones that might actually arise, and which therefore would generate results that might have little applicability in the real world. Although an overall requirement that the potential benefits justify the risks remains, without the missing principle, highly speculative research might be judged as having benefits that outweigh an arbitrary amount of risk. Moreover, like earlier U.S. government policies, the 2024 policy does not capture research done without government funding. Extending oversight to similar research conducted without government support would require new regulations to be issued under existing statutory authority, or the passage of new legislation. However, this step has not yet been taken.

To date, only four research projects to date have been put through these oversight frameworks, a surprising result that is inconsistent with survey data collected from biosafety professionals about how much research PEPP research is currently underway. This discrepancy should be resolved. It remains to be seen to what extent future research projects will be identified for review and whether the review mechanism will be able to keep up with the number of proposals that are referred to it. Conversely, if the number of projects to review remains at very low levels (say, less than one per year), either additional guidance may be necessary to ensure that researchers and research institutions are appropriately referring PEPP research for review, or the “trigger language” of the PEPP policy might be reviewed to see if it should be broadened to capture additional proposals that may merit further review but currently are not getting it.

## Notes

^a^
Certain potential pandemic viruses, including the SARS coronavirus (now known as SARS-COV-1) and reconstructed viable forms of the 1918 influenza virus, were controlled under the Federal Select Agent Program, which regulates possession, transfer, and use of pathogens designated by the U.S. government as being threats to public health and safety. However, no policies were in place to govern research with potential pandemic pathogens in general.

^b^
Use of “blue sky” here draws on the idiom “bolt from the blue,” referring to a lightning strike coming from a “blue sky” with no nearby thunderstorm. A “bolt from the blue” is a completely unexpected and very surprising occurrence.

^c^
The fact that government agencies that did not have their own P3CO review policies were still bound by the 2014 funding pause was apparently not necessarily known by all funding officers in other agencies with whom P3CO policies were discussed. Personal communication, Thomas Inglesby, Johns Hopkins Center for Health Security

^d^
Based on the author’s experience as chair of the interagency committee responsible for producing the 2017 OSTP P3CO Guidance.

^e^
EcoHealth Alliance’s lack of compliance with this condition was an element in its later suspension and debarment from federally funded programs.

^f^
Public Law 117–328. Division FF, Title II

^g^
IBBIS’ initial work is focusing on mitigating risks from DNA synthesis technology. See https://ibbis.bio.
